# Investigating the Increasing Azole Resistance in Candida Infections Among Critically Ill Patients: Experience From a Tertiary-Level Setup in North India

**DOI:** 10.7759/cureus.98425

**Published:** 2025-12-03

**Authors:** Sweta Singh, Sana Islahi, Kalicharan Das, Shefali Gupta

**Affiliations:** 1 Clinical Microbiology, All India Institute of Medical Sciences, Raebareli, IND; 2 Microbiology, All India Institute of Medical Sciences, Raebareli, IND; 3 Neuroanesthesiology and Critical Care, All India Institute of Medical Sciences, New Delhi, IND

**Keywords:** antifungal stewardship, antifungal susceptibility, azole resistance, candida species, echinocandins, minimum inhibitory concentration, non-albicans candida

## Abstract

Introduction: Candida species are among the most common fungal pathogens worldwide, with varied clinical presentations and manifestations in both immunocompetent and compromised individuals. In recent years, both global and Indian research have documented a progressive shift from *Candida albicans *to treatment-resistant non*-*albicans Candida (NAC)spp. Compared with Western countries, there is a dearth* *of information on invasive Candida infections in India. The azole group of drugs is most widely used for Candida infections, and this is the reason for the increasing resistance reported in this class of drugs. Recently, resistance to azoles has increased in Candida species, both in clinical settings and in vitro.

Objectives: The present study aims to identify the spectrum of Candida species in clinical infections and to identify their sensitivity pattern to available antifungal agents.

Methods: The study was conducted over 24 months, from April 2023 to March 2025, on clinical samples received for routine culture and sensitivity testing. Antifungal sensitivity was tested according to the Clinical and Laboratory Standards Institute guidelines and interpreted. Demographic and other patient parameters were also assessed.

Statistical analysis: The results were analyzed using the Statistical Package for the Social Sciences version 22 software (SPSS Inc., Chicago, IL).

Results: The total number of samples analyzed for fungal etiology during the specified time period was 382, of which 57.5% (n = 219) were contributed by blood, 29.2% (n = 112) by urine samples, and the remaining 13.3% (n = 51) by intra-abdominal samples, tissue, pus, and body fluids. Individually, *C. albicans* was higher in blood samples than in urine samples (43.5% vs. 41.7%)*.* The overall resistance rates in blood isolates to fluconazole and voriconazole were reported as 21.4% and 5.6%, respectively. The trend of Azole resistance from 2023 to 2025 increased for fluconazole from 18.5% to 34.6% and for voriconazole from 5.7% to 10.2%. Echinocandin resistance, however, was not notable, ranging from 0.6% in 2023 to 1.6% in 2025.

Conclusion: Our study emphasizes the role of increased Candida isolates and resistance trends across various samples over the specified time period. *C. albicans *was the most commonly isolated Candida species overall, but the gradual shift of Candida species to non-albicans species (NAC) is very much pertinent in our study. *Candida auris *remains undetected in blood and other invasive samples in the present study. Our study did not report any *C. auris *isolate. This may be due to the constant and strong reinforcement of enhanced infection control measures, including environmental decontamination and contact precautions. The present study showed a significant increase in antifungal susceptibility testing volumes over the study period, reflecting the growing clinical burden of Candida species. The potential rise in the azole group of drug resistance emphasizes the need for continued regional surveillance to track resistance trends.

Expanding regional and global surveillance efforts and developing novel antifungal agents are crucial to addressing the challenges posed by rising multidrug-resistant Candida isolates in various clinical samples. Enhanced surveillance, antifungal stewardship programs, and innovation are very much essential to mitigate the public health impact of antifungal resistance and improve the clinical outcomes among patients.

## Introduction

Apart from bacterial infections, fungal infections also play a major role in negative patient outcomes in debilitated individuals. Candida species are among the most common fungal pathogens worldwide, with varied clinical presentations and manifestations in both immunocompetent and compromised individuals [1].

Candida species are part of the normal colonizers of the oral cavity, gastrointestinal tract, and vaginal tract. They can cause varied manifestations ranging from mucocutaneous to bloodstream infections (BSIs). The incidence of Candidemia reported in several studies approximates 6.9 per 1,000 critically ill patients, and 7.5% of these patients were on antifungal therapy [2,3].

Candidemia has alarming mortality rates of 20%-49%. Studies on invasive candidiasis report prevalence rates of 10% in BSIs and 25% in urinary tract infections [4]. Candida, being the normal colonizer of the vaginal tract in females, gives rise to vulvovaginal candidiasis, which occurs once in their lifetime in almost 75% of all women. Apart from bacterial causes of nosocomial BSIs, Candida also remains one of the most important causes of BSIs. Candida-induced BSIs carry a much higher rate of mortality, ranging up to 72% [4]. Immunocompromised states with a history of intubation and central venous catheter insertion are major risk factors for invasive Candida infections. Both albicans and non-albicans Candida (NAC) species are increasingly being reported in various clinical specimens in recent years.

More than 90% of invasive infections are caused by *Candida albicans*, *Candida glabrata*, *Candida parapsilosis*, *Candida tropicalis*, and *Candida​​​​​​​ krusei* [5]. The potential clinical importance of species-level identification lies in the fact that Candida species differ in the expression of putative virulence factors and antifungal susceptibility patterns. In recent years, both global and Indian research have documented a progressive shift from *C. albicans* to treatment-resistant NAC spp. A substantial knowledge gap exists regarding the prevalence rates of invasive Candida infections from this subcontinent compared to other developed countries [6,7].

Literature search of the past few decades highlights the increased isolation of azole-resistant Candida species like *C. parapsilosis* and *C. auris*. Fluconazole resistance is increasingly being noted in *C. parapsilosis* isolates in various clinical settings [8]. Invasive candidiasis has been on the rise in recent years during and after COVID-19, and this may be attributed to the increasing immunocompromised states due to malignancies, use of steroids, longer hospital stay, etc. The azole group of drugs is most widely used for Candida infections, and this is the reason for the increasing resistance reported in this class of drugs.

Rising resistance to the azole group of antifungals, both in inpatients and community settings, is a cause of concern in recent times. The mechanism of action of the azole group of antifungals is by interfering with the enzyme lanosterol 14-α-sterol demethylase, which is involved in ergosterol biosynthesis. This ergosterol forms the major component of the fungal cell wall, which gets disrupted once the enzyme lanosterol 14-α-sterol demethylase is interfered with. Overall, this leads to the alteration in the functioning of the cell membrane and inhibition of fungal growth [9].

There are three main mechanisms of azole resistance in Candida species. Multidrug pumps may get introduced inside the fungal cell wall, and this leads to the pumping of the drug outside, which in turn decreases the inhibition of the enzyme lanosterol 14-α-sterol demethylase. Second, it may be due to upregulation of the ERG11 gene, which alters the enzyme's binding site and renders the azole ineffective. Finally, mutations may lead to the formation of bypass pathways, which may prevent the accumulation of toxic by-products and allow the fungus to retain its normal functional cell membrane [10]. The emergence of drug-resistant isolates of Candida species will have an overall impact on poorer clinical outcomes in the patient as well as increased health care costs [11].

The present study was designed to investigate the varied spectrum of infections caused by various Candida species and also to assess their antifungal sensitivity profiles. This study will serve as a benchmark for the study of alarming rise in resistance for the azole group of drugs in Candida isolates in a tertiary level set up of North India. This also necessitates the formulation of proper antifungal stewardship strategies in hospitals to target antifungal resistance in this region of the country and prevent clinical outbreaks.

## Materials and methods

It was a retrospective cross-sectional study conducted from April 2023 to March 2025 (24 months) on clinical samples received for routine culture and sensitivity testing in the Mycology lab of the department from hospitalized, critically ill patients across different wards of our institute.

The primary objective is to estimate the prevalence and trends of azole resistance in different Candida isolates in critically ill patients, and the secondary objectives are to determine the demographic pattern and associated risk factors of azole-resistant Candida isolates and to assess the resistance pattern and outcomes associated with different Candida species resistant to azole antifungals.

All the clinical samples received for routine culture and sensitivity in the Mycology lab of the department were included in the study. Out of these, azole-resistant Candida isolates were further assessed for prevalence, demographic parameters, risk factors, and resistance profile. Duplicate samples from the same patient on the same day showing similar culture positivity were excluded from the study.

Clinical samples, such as urine, pus, sputum, blood, endotracheal secretions, cerebrospinal fluid, and other body fluids received in the section of Mycology for routine fungal culture and sensitivity, were included in the study. All the samples were cultured on routine media as well as on chromogenic media for early species identification. CHROM Candida agar and biochemical tests were used to identify the species. Routine antifungals tested in the lab were azole drugs (fluconazole and voriconazole), polyenes (amphotericin B), and echinocandins (caspofungin, anidulafungin, and micafungin). Antifungal sensitivity was tested according to the Clinical and Laboratory Standards Institute (CLSI) guidelines and interpreted [9,10]. Demographic and various other patient parameters were also assessed.

The Hospital Management Information System was used to extract demographic and clinical parameters for patients, along with patient records from various wards. The medical records were later deidentified.

Collected data were computerized and statistically analyzed using the Statistical Package for the Social Sciences version 22 program (IBM Corp., Armonk, NY). Qualitative data were represented as percentages. Accuracy was measured using sensitivity, specificity, positive predictive value, and negative predictive value. The chi-square test was used to assess differences between qualitative variables. A p value of <0.05 was used for statistically significant values and results.

## Results

Sample distribution and species frequency

**Figure 1:**
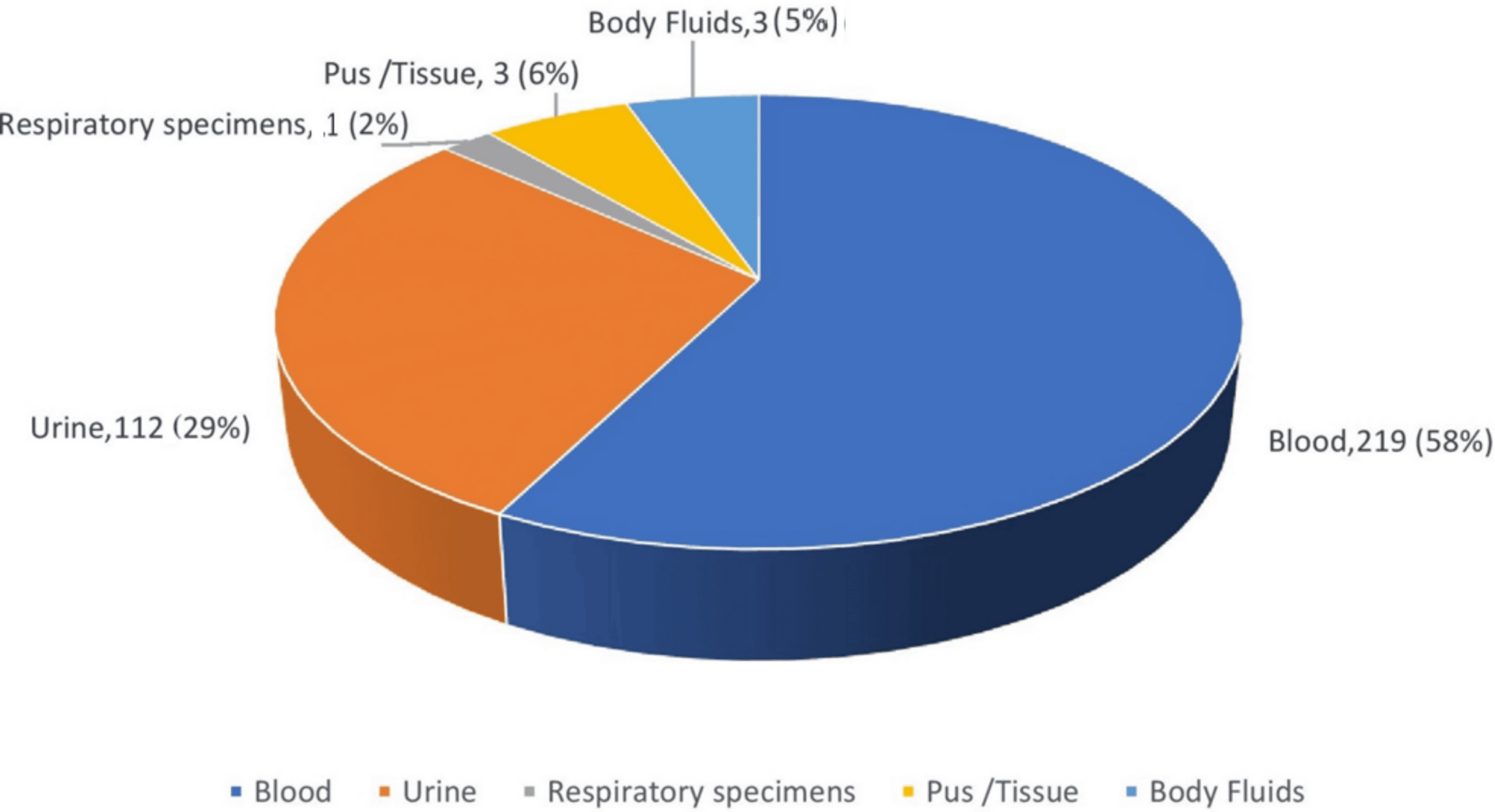
Sample distribution for yeast isolates Overall distribution of samples during the study period in percentage and actual numbers

Three hundred eighty-two total samples were analyzed for fungal etiology. Among these, n = 219 (57.5%) was attributed by blood, n = 112 (29.2%) by urine samples, and the remaining, n = 51 (13.3%) by intra-abdominal samples, tissue, pus, and body fluids. Sample distribution for yeast isolates is depicted in Figure 1. Overall, culture positivity for yeast isolates was observed at n = 118 (31%). Among the culture-positive cases, the majority were attributed to urine and blood specimens. Antifungals were prescribed for yeasts isolated in the urine of a selected group of patients, such as neutropenic patients, very low-birth-weight infants (<1,500 g), and patients who were to undergo urological procedures or surgeries.

**Figure 2:**
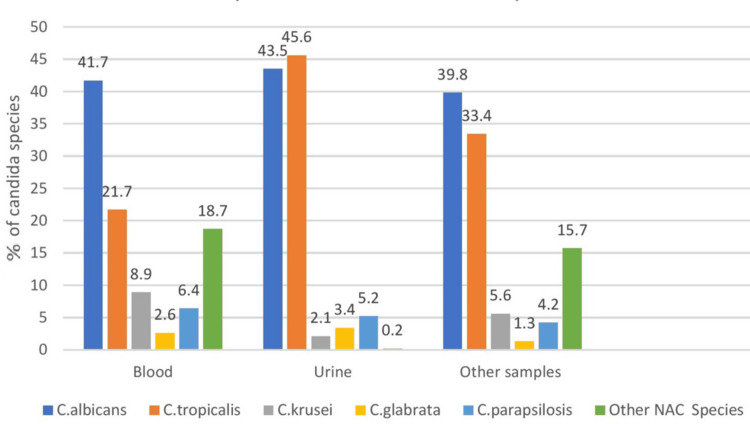
Species distribution in various samples Distribution of various Candida species in the specified time period

The majority of clinical specimens (113/118) were positive for a single isolate each, but 4.3% of patients (5/118) yielded two or more isolates. The frequency of the various species is depicted in Figure 2. The proportions of *C. albicans* and *C. tropicalis* were higher in blood and urine cultures than in the other Candida species. Individually, *C. albicans* was higher in blood samples compared to urine samples, n = 51 (43.5%) vs. N = 49 (41.7%). *C. tropicalis* was the second most abundant isolate in urine samples after *C. albicans*, n = 54 (45.6%) vs. n = 51 (43.5%). Among the other prominent Candida species isolated were *C. glabrata*, *C. parapsilosis*, *C. krusei*, etc. We did not isolate any *C. auris* in any of our tested samples.

Antifungal resistance rates found in various isolates

**Figure 3:**
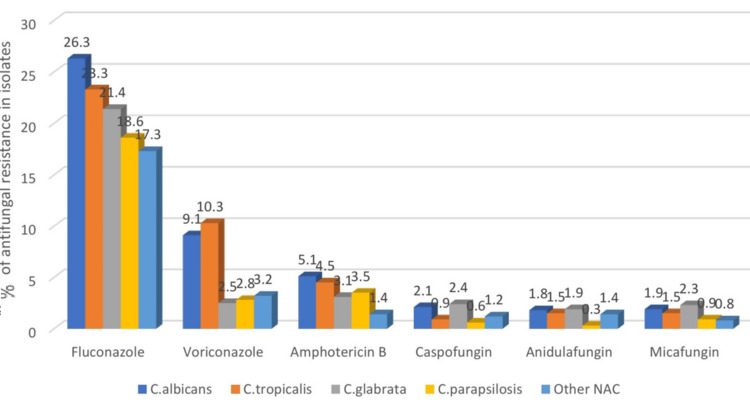
Antifungal resistance rates for blood isolates Resistance pattern to azole, amphotericin B, and Echinocandin drugs in blood samples

The overall resistance rates in blood isolates for fluconazole were reported as 25/118 (21.4%) and voriconazole as 7/118 (5.6%) (Figure 3). Further categorization according to the Candida species, the authors found that *C. albicans* showed 31/118 (26.3%) resistance rates, *C. tropicalis* 27/118 (23.3%), *C. glabrata* 25/118 (21.4%), and *C. parapsilosis* 22/118 (18.6%). In contrast, other NAC species had 20/118 (17.3%) resistance to fluconazole. Likewise, speaking of voriconazole resistance: *C. albicans* showed 11/118 (9.4%), *C. tropicalis* 12/118 (10.3 %), *C. glabrata* 3/118 (2.5%), *C. parapsilosis* 3/118 (2.8 %), whereas other NAC species had 4/118 (3.2%) resistance.

**Figure 4:**
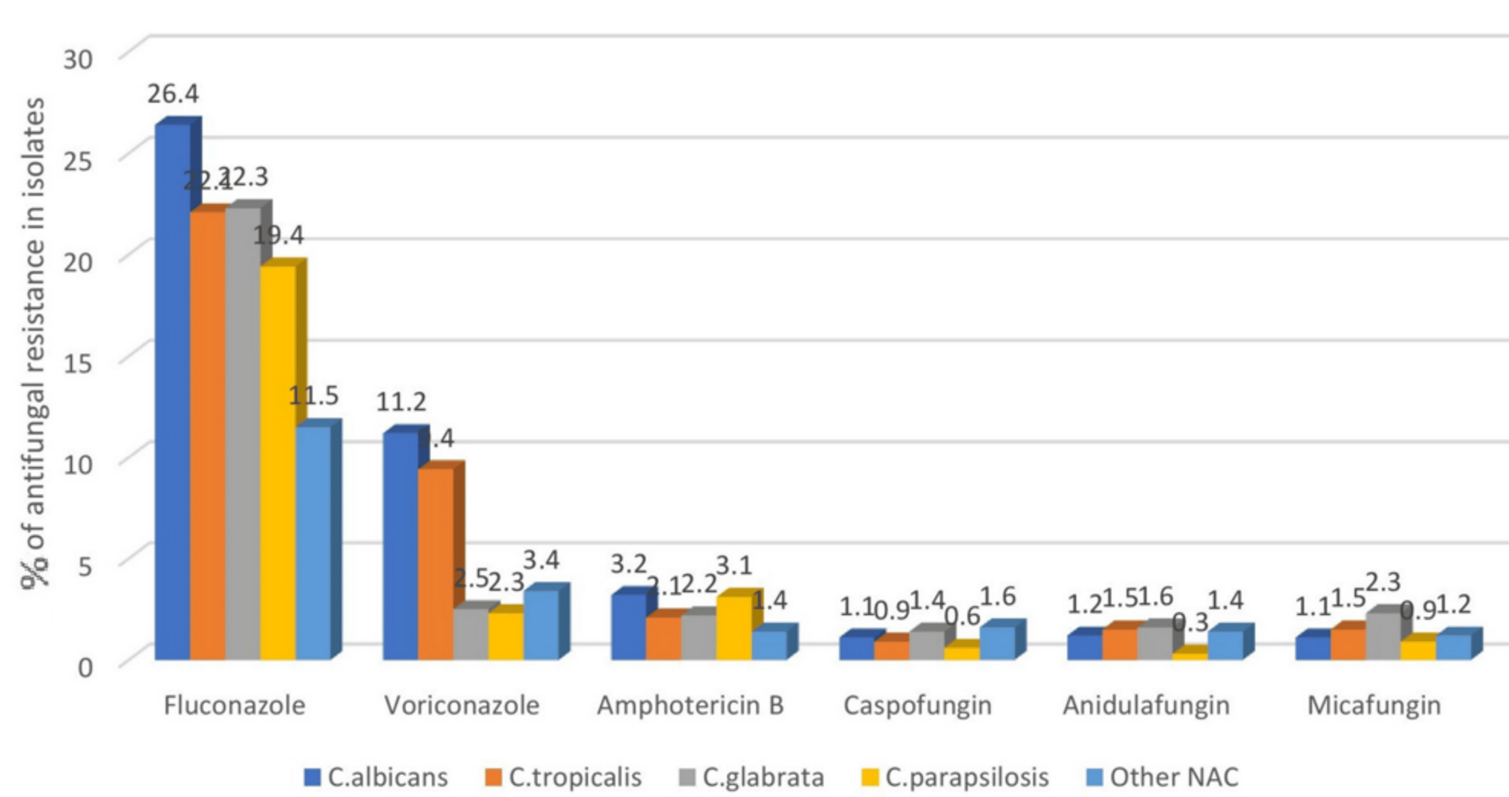
Antifungal resistance rates for non-blood isolates Resistance pattern to azole, amphotericin B, and echinocandin drugs in urine, pus, and miscellaneous samples

The overall resistance rates in non-blood isolates for fluconazole were reported as 24/118 (20.3%), and for voriconazole as 7/118 (5.7%) (Figure 4). Further categorization according to the Candida species, the authors found that *C. albicans* showed 31/118 (26.4%) resistance rates, *C. tropicalis* 26/118 (22.1%), *C. glabrata* 26/118 (22.3%), and *C. parapsilosis* 23/118 (19.4%), whereas other NAC species had 13/118 (11.5%) resistance to fluconazole. Likewise, speaking of voriconazole resistance: *C. albicans* showed 13/118 (11.2%), *C. tropicalis* 11/118 (9.4%), *C. glabrata* 3/118 (2.5%), *C. parapsilosis* 3/118 (2.3%), whereas other NAC species had 4/118 (3.4%) resistance.

These findings in blood and non-blood isolates yielded no significant difference between the resistance rates to the azole group of drugs. Amphotericin B showed much higher sensitivity and resistance rates of less than 2% in both types of samples. The echinocandin group of drugs still managed to be the most effective group of drugs for the blood and non-blood isolates, with resistance rates of less than 1%. Species-wise, *C. albicans* and *C. tropicalis* showed maximum resistance to the azole group of drugs in both types of isolates.

Trends of resistance to azole antifungal drugs over time

**Figure 5:**
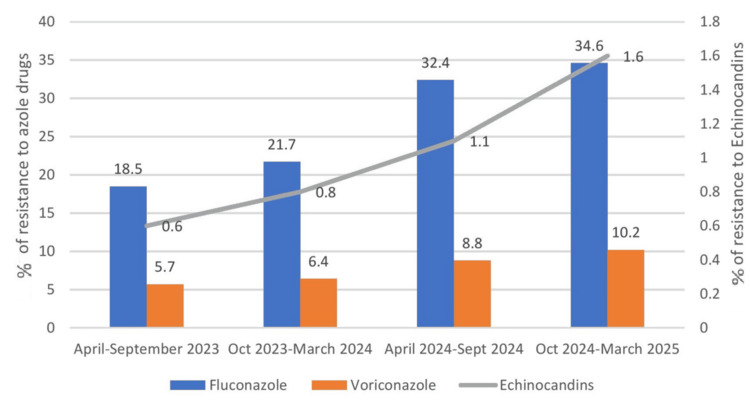
Trend of resistance to azole antifungals The trend of azole resistance from 2023 to 2025 increased for fluconazole and voriconazole. Overall, echinocandin resistance rates were low and steady, regardless of the sample type

The trend of azole resistance from 2023 to 2025 increased for fluconazole from n = 7/36 (18.5%) to n = 28/82 (34.6%); and for voriconazole from n = 2/36 (5.7%) to n = 8/82 (10.2%). Echinocandin resistance, however, was not notable, ranging from 1/36 (0.6%) in 2023 to 2/82 (1.6%) in 2025. Overall, echinocandin resistance rates were low and steady, regardless of the sample type (Figure 5).

**Figure 6:**
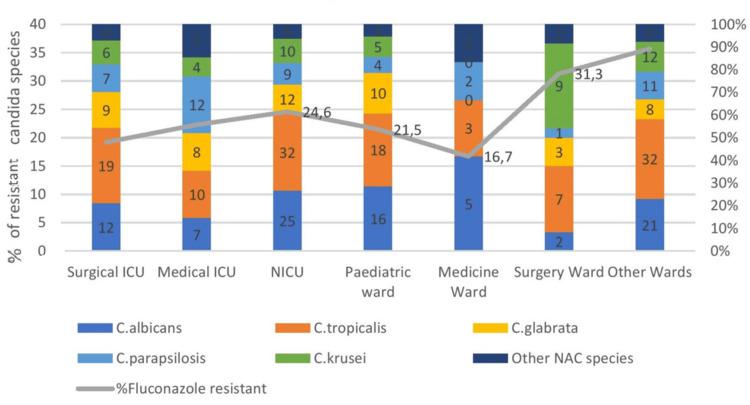
Categorization as per admission ward and species with their resistance rates Epidemiology of Candida spp. from the blood cultures of inpatient wards. C. albicans accounted for n = 65/118 (54.9%) of isolates, being the dominant species in most wards, except for the following wards: NICU, pediatrics, Medical ICU, Surgical ICU, etc. C. tropicalis showed dominance in pediatric wards and NICU NICU: neonatal intensive care unit; NAC: non-albicans Candida

Fluconazole resistance is increasingly being noted in *C. parapsilosis* isolates in various clinical settings. This is reflected by the rates of isolation in the study period as follows: 2023 (n = 2 (3.2%)) < 2024 (n = 4 (11.0%)) < 2025 (n = 12 (20.6%)) (p < 0.05). On the contrary, if we exclude the *C. parapsilosis* isolates showing resistance to fluconazole, this resulted in lower azole resistance rates: 2023 (n = 3 (5.3%)), 2024 (n = 2 (6.1%)), 2025 (n = 1 (4.8%)) (p < 0.05). Hence, isolation of non-albicans fluconazole-resistant Candida species over the years has a significant association with increasing azole resistance in Candida isolates (Figure 6).

Trends in species epidemiology in blood cultures as per ward of admission over time

From the advent of 2023 and stepping into 2025, the authors noticed a significant shift in the trend from the albicans Candida species to the NAC species. From 2023 to 2025, in blood cultures, we observed increasing proportions of NAC: 2023 (n = 11 (24.6%)) < 2024 (n = 17 (30.5%)) < 2025 (n = 24 (38.1%)) (p < 0.05); and decreasing proportions of *C. albicans:* 2023 (n = 17 (51.6%)) > 2024 (n = 12 (43.1%)) > 2025 (n = 18 (29.8%)) (p < 0.05). The authors also noticed a gradual increase in *C. glabrata* after the COVID-19 pandemic from 2023 to 2025: 2023 (n = 4 (11.7%)) > 2025 (n = 9 (19.8%)) (p < 0.05); and a decrease in other Candida spp. in 2023 compared with 2025 (p < 0.05).

Species distributions in the present study were ward-dependent (Figure 6). The authors studied the epidemiology of Candida spp. from the blood cultures of inpatient wards. *C. albicans* accounted for n = 65/118 (54.9%) of isolates, being the dominant species in most wards, except for the following wards: NICU, Pediatrics, Medical ICU, Surgical ICU, etc. *C. tropicalis* showed dominance in pediatric wards and NICU. *C. glabrata *was also increasingly isolated in the wards, especially in pediatric wards and NICU wards, n = 11 (23.6%), but undetected in the Medicine ward. *C. parapsilosis* proportions were particularly high in the NICU, Medical ICU, and Surgical ICU; n = 21 (18.3%), and accounted for n = 11-18, 5.0%-6.2% in other wards. Finally, *C. krusei* and other Candida spp. were particularly frequent in the NICU and other wards, with n = 6 and n = 8 (8.4% and 13.4%, respectively).

Comparison of comorbidities and clinical outcomes in the two groups: azole-sensitive and azole-resistant Candida infections

Azole resistance showed a significant association with immunocompromised states, namely, malignancies (both organ and hematological; p < 0.001) and chronic conditions like diabetes (p < 0.005). Predictors of poor outcome in patients were also significantly associated with azole resistance, such as intubation (p < 0.005). In contrast, other parameters, such as use of vasopressor drugs/medications (p = 0.233) and ventilatory support (p = 0.123), had no significant association with poor patient outcomes when comparing the two groups. Furthermore, this all added to poor patient outcomes, which was reflected by a significant association in inpatient mortality rates between azole-sensitive and resistant cases (p < 0.00001) (Table 1).

**Table 1:** Comparison of comorbidities and clinical outcomes in the two group: azole-sensitive and azole-resistant Candida infections OR: odds ratio; COPD: chronic obstructive pulmonary disease; CKD: chronic kidney disease; CLD: chronic liver disease

Clinical parameters	Azole-sensitive Candida infections (n = 300, 78.6%)	Azole-resistant Candida infections (n = 82, 21.4%)	OR value	Chi-square statistic	p values
Age (mean, range in years)	68 (8-75)	59 (3-71)	0.04-0.05	2.0534	0.151
Males	136	38	0.00-0.01	0.0264	0.019
Females	164	44	0.00-0.01	0.0264	0.019
Hypertension	91	8	0.00-0.04	0.0388	0.560
Diabetes mellitus	31	77	2.14-0.61	7.3694	0.006
COPD/asthma	14	2	0.03-0.27	0.0388	0.712
CKD	12	6	0.11-0.30	1.0227	0.421
CLD	10	2	0.16-0.45	1.0277	0.311
Heart diseases	5	1	0.88-1.08	2.0534	0.151
Malignancies (organ/hematological)	7	57	3.60-1.02	6.5694	<0.001
In-patient mortality	21	45	6.76-8.50	21.851	<0.00001
Use of ventilators	24	98	0.90-0.28	2.3741	0.123
Use of vasopressive drugs	34	87	0.91-0.28	3.4351	0.233
Intubation	101	52	2.92-3.67	21.851	<0.005

## Discussion

Our study emphasizes the role of increasing Candida isolates as well as resistance trends in various samples over the specified time period. *C. albicans* was the most commonly isolated Candida species overall, but the gradual shift of albicans species to non-albicans species (NAC) is very much pertinent in our study. We could not isolate any *C. auris* in our present study, which may be due to the lack of typing and sequencing strains for this particular species.

The proportions of *C. albicans* and *C. tropicalis* were higher in blood and urine cultures compared to the other Candida species. Individually, *C. albicans* was higher in blood samples than in urine samples (43.5% vs. 41.7%). *C. tropicalis* was the second most abundant isolate in urine samples after *C. albicans* (45.6% vs. 43.5%). Among the other prominent Candida species isolated were *C. glabrata*, *C. parapsilosis*, *C. krusei*, etc. The distribution of Candida species in isolates collected during the study period, in both blood cultures and other samples, was very much comparable to previously done studies [12,13].

The trend of azole resistance from 2023 to 2025 increased for fluconazole from 18.5% to 34.6% and for voriconazole from 5.7% to 10.2%. Echinocandin resistance, however, was not notable, ranging from 0.6% in 2023 to 1.6% in 2025. The echinocandin group of antifungals maintained good sensitivity rates for various isolates of Candida. This is in concordance with similar studies in other regions [14,15]. Fluconazole resistance is increasingly observed in *C. parapsilosis* isolates across various clinical settings. This is reflected by the rates of isolation in the study period as follows: 2023 (3.2%) < 2024 (11.0%) < 2025 (20.6%) (p < 0.05). On the contrary, if we exclude the *C. parapsilosis* isolates showing resistance to fluconazole, this resulted in lower azole resistance rates: 2023 (5.3%), 2024 (6.1%), and 2025 (4.8%) (p < 0.05). Hence, isolation of non-albicans fluconazole-resistant Candida species over the years has a significant association with increasing azole resistance in Candida isolates. A similar study conducted in European countries by Arendrup et al. revealed a high fluconazole resistance rate of 17% in *C. parapsilosis* blood isolates. Similar studies done worldwide in Greece, Turkey, and Italy detected fluconazole resistance rates of 37%, which is very much in line with our finding of azole resistance in Candida isolates [15]. Echinocandins have a lower sensitivity profile for *C. parapsilosis*; hence, the emergence of azole resistance in this group is a matter of grave concern for clinical settings.

Our clinical setting is a growing tertiary care institute in the said area, and the authors noticed a substantial increase in the testing volumes of antifungal sensitivity in the studied time period. This is a clear indication of the rising burden of invasive Candida infections in clinical settings. This alarming rise in the resistance toward the azole group of drugs warrants the need for strong antifungal stewardship plans along with active regional surveillance.

CDC reported that about 7% of all Candida-positive blood samples were resistant to fluconazole. This study agrees with our findings due to the increasing isolation of fluconazole-resistant Candida species in *C. parapsilosis*, *C. auris*, etc.

Since the advent of 2018, various severe outbreaks have been documented and studied worldwide with fluconazole-resistant *C. parapsilosis* [16,17]. Likewise, different studies have highlighted the alarming rise in fluconazole-resistant *C. tropicalis* in both urine and invasive samples [18,19]. *C. glabrata* and some other species of Candida have exhibited greater variability in susceptibility to both azoles and the echinocandins, a group of antifungal drugs, which makes individualized susceptibility testing the need of the hour to guide optimal antifungal therapy.

Our study did not report any *C. auris* isolate. This may be due to the constant and strong reinforcement of infection control practices in our hospital setting in the form of proper decontamination measures and standard precautions [20].

The present study additionally tried to assess the clinical outcomes and patient-specific factors that helped to correlate the resistance patterns with treatment efficacy. Predictors of poor outcome in patients were also significantly associated with azole resistance, such as intubation (p < 0.005). In contrast, other parameters, such as use of vasopressor drugs/medications (p = 0.233) and ventilatory support (p = 0.123), had no significant association with poor patient outcomes when comparing the two groups. Furthermore, this all added to poor patient outcomes, which were reflected by a significant association in inpatient mortality rates between azole-sensitive and resistant cases (p < 0.00001) [21,22].

Our study had a few limitations. First, this was a retrospective study conducted in our single hospital setting, so the findings will not fully reflect other geographical areas. Second, there are no definitive guidelines or documents for the minimal inhibitory concentration interpretation of certain Candida species, like *C. glabrata* and *C. auris*. They require extracellular volume values for interpretation and are not fully applicable in clinical scenarios. There were limited data available for previous fungal use, as previous hospitalization and therapy could not be documented.

Despite these limitations, the present study provides useful data and insights into the increasing resistance to commonly used antifungal drugs. This study also strengthens the need for active surveillance and targeted intervention, especially in this region of the country. This alarming rise in the resistance toward the azole group of drugs warrants the need for strong antifungal stewardship plans along with active regional surveillance. The integration of advanced molecular techniques, such as whole-genome sequencing, will help add novelty to the list of antifungals and improve diagnostic modalities [23,24].

Antifungal stewardship programs are the need of the hour and are essential to mitigate the development of antifungal resistance. The future lies in exploring novel adjunctive therapies for invasive Candida infections, such as immunomodulators, to win the war against antifungal resistance [24].

## Conclusions

This retrospective study provides clear and critical insights into the alarming rates of increasing azole resistance in Candida isolates. While echinocandins still prove to be efficacious for *C. albicans*, *C. parapsilosis*, and *C. tropicalis*, increasing fluconazole resistance warrants continued surveillance.

Continued efforts toward regional as well as global surveillance to track the increasing resistance rates is the need of the hour. Addition of a novel antifungal agent in the current limited antifungal armamentarium is very much essential to mitigate the effects of increasing multidrug-resistant Candida infections and improve patient outcomes. *C. glabrata* due to its significant variability in susceptibility patterns warrants the need for individualized testing and treatment strategies.
